# Viral Infection: An Evolving Insight into the Signal Transduction Pathways Responsible for the Innate Immune Response

**DOI:** 10.1155/2012/131457

**Published:** 2012-09-11

**Authors:** Girish J. Kotwal, Steven Hatch, William L. Marshall

**Affiliations:** ^1^University of Medicine and Health Sciences, St. Kitts, New York, NY 10001, USA; ^2^Division of Infectious Disease and Immunology, Department of Medicine, University of Massachusetts Medical School, 364 Plantation Street, Worcester, MA 01605, USA

## Abstract

The innate immune response is initiated by the interaction of stereotypical pathogen components with genetically conserved receptors for extracytosolic pathogen-associated molecular patterns (PAMPs) or intracytosolic nucleic acids. In multicellular organisms, this interaction typically clusters signal transduction molecules and leads to their activations, thereby initiating signals that activate innate immune effector mechanisms to protect the host. In some cases programmed cell death—a fundamental form of innate immunity—is initiated in response to genotoxic or biochemical stress that is associated with viral infection. In this paper we will summarize innate immune mechanisms that are relevant to viral pathogenesis and outline the continuing evolution of viral mechanisms that suppress the innate immunity in mammalian hosts. These mechanisms of viral innate immune evasion provide significant insight into the pathways of the antiviral innate immune response of many organisms. Examples of relevant mammalian innate immune defenses host defenses include signaling to interferon and cytokine response pathways as well as signaling to the inflammasome. Understanding which viral innate immune evasion mechanisms are linked to pathogenesis may translate into therapies and vaccines that are truly effective in eliminating the morbidity and mortality associated with viral infections in individuals.

## 1. Introduction

The innate immune system is as ancient as the bacterial immune response to bacteriophages. As the nature and complexity of viral innate immune evasion mechanisms evolved, so has the innate—and eventually adaptive—immune response to these mechanisms. The innate immune response in mammals is initiated by the interaction of stereotypical pathogen components with germ-line encoded receptors. In some cases, signal transduction pathways are stimulated in sentinel cells, such as macrophages and dendritic cells. Stimulation of these signaling pathways promptly activates innate effector mechanisms to protect the host; these innate immune signals also activate antigen-presenting cells that are critical to the eventual adaptive immune response of the host [1]. In this paper we will summarize findings in the innate immune system that are relevant to viral pathogenesis and outline the evolution of viral mechanisms that suppress innate immunity in mammalian hosts.

## 2. The Innate Immune System

The receptors of the innate immune system are germ-line encoded and include the nucleotide-binding domain leucine-rich repeat containing receptors, the Toll-like receptors (TLRs), and the RIG-I-like receptors (RLRs). The RLRs are cytosolic sensors of pathogen RNA and include proteins encoded by the retinoic acid-inducible gene-I (RIG-I) [2], the melanoma differentiation-associated gene 5 (MDA5) [3, 4], and the laboratory of genetics protein 2 (LGP2) [4] and DDX3, which is thought to associate with RIG-I [5]. The helicase domains of RLRs detect the cytosolic RNA of microbial pathogens, generating signals that drive production of cytokines and interferons. Helicases are ATP-dependent enzymes that unidirectionally translocate along a transcript thereby dissociating nucleic acid duplexes [6]. The RIG-I and MDA5 RLRs play critical roles in the recognition of foreign RNA and in the response to many viral pathogens. MDA5 and RIG-I contain a DExD/H-box RNA helicase domain and caspase activation and recruitment domains (CARDs). RIG-I recognizes 5′-triphosphate RNA, and MDA5 can recognize complex webs of pathogen RNA, comprised of both viral single-stranded and double-stranded RNA [2]. The LGP2 RLR protein was found to lack a CARD domain and was originally identified as a dominant negative inhibitor of RIG-I signaling [7]. Under some circumstances, though, it appears LGP2 can stimulate RLRs such as MDA5 and RIG-I [8]. CARD engagement leads to interaction with a protein known as mitochondrial antiviral signaling protein (MAVS) that is alternatively designated CARDIF, HELICARD, or IPS-1 (referred to here as MAVS) [9, 10]. Subsequently, upon oligomerization, MAVS signals to members of the IKK family of kinases that are critical for the innate immune response [10]. Thus MAVS induces IKK*α* and IKK*β* stimulation that leads to translocation of NF-*κ*B, as well as IKK*ε*/TBK1 stimulation that leads to translocation of IRF-3. These transcription factors stimulate production of cytokines, other innate immune response proteins, and type I interferons [4].

Extracytosolic innate immune sensing of pathogens is mediated via the TLRs. Humans are known to encode ten TLRs which are each involved in the recognition of different pathogen-associated molecular constituents [11]. The TLRs are transmembrane receptors found on the cell surface and/or associated with endocytic vesicles [11]. Thus, they are ideally situated to detect extracytosolic pathogens. For example, TLR4 is required for the recognition of Gram-negative bacterial lipopolysaccharide (LPS, or endotoxin) while TLR3 is able to recognize dsRNA, a signature compound common in the lifecycle of many viruses, while TLR7 and 8 recognize ssRNA [12]. Toll/IL-1 interacting receptor (TIR) adapters ultimately stimulate IkB family kinases (IKKs) often via transducing proteins such as IRAKs and TRAFs, thereby mediating signaling originally induced by engagement of TLRs that ultimately activates NF-*κ*B and IRF3 [13].

Two classical IKKs, IKK*α* and IKK*β*, are critical for NF-*κ*B activation. They function, in large part, by phosphorylating the inhibitors of NF-*κ*B, known as IkBs. Once phosphorylated, IkBs are ubiquitinated and degraded. This allows NF-*κ*B subunits to translocate to the nucleus and activate target gene expression. NF-*κ*B is critical for driving the expression of numerous cytokines, chemokines, and costimulatory molecules, creating an inflammatory response [14]. On the other hand, the two nonclassical IKK family members, IKK*ε* and TBK1, are implicated in IRF3 activation. In particular, they are believed to directly phosphorylate several serine residues within the C-terminal activation domain of IRF3. Once phosphorylated, IRF3 dimerizes and translocates to the nucleus where it activates target gene expression [15]. IRF3 activation is critically important for the activation of type I interferons, either directly [16] or via an autocrine/paracrine loop [17, 18]. Type I interferons, in turn, are capable of inducing a significant antiviral response in the host [2, 13, 19].

Cells also encode cytosolic DNA sensors which detect DNA, which is not typically present in the cytosol and thus a pattern whose recognition signals the presence of viral nucleic acids. Nucleotide oligomerization domain-like receptor proteins (NLRs) are implicated in the intracytosolic recognition of sterile inflammatory instigators, such as urate crystals, intracytosolic DNA, or viral RNA. One such NLR, nucleotide oligomerization domain-like receptor protein 3 (NLRP3) is an inflammasome component that signals to the apoptosis-associated speck-like protein containing a caspase recruitment domain (ASC) to induce the clustering-induced self-processing of procaspase 1 into caspase 1 which then digests the precursor form of pro-IL-1*β* and pro-IL-18 to permit release of the inflammatory cytokines IL-1*β* and IL-18 from the cell [20]. Similar inflammatory pathways are triggered by engagement of other cytosolic DNA sensors such as AIM-2, another NLR that ultimately induces cleavage of procaspase 1 into caspase 1. Aim-2 can detect the molecular patterns of intracellular hazards such as pathogen DNA, particularly that of poxviruses [21]. Other NOD proteins which are alternatively designated NACHT, LRR, and PYD function as sensors of toxic intracellular molecules including cytosolic DNA [22]. Thus the NLRs represent examples of cytosolic DNA sensors capable of inducing an inflammatory antiviral response.

Another antiviral cytosolic sensor is the DNA-dependent activator of interferon (DAI), which binds B- and Z-form DNA, thereby recognizing intracytosolic viral DNA. Signals from such sensors are transduced by known innate immune kinases such as TBK1, which interacts with a protein known as stimulator of interferon genes (STINGs) to activate NF-*κ*B and IRF3 signaling [10, 23, 24]. Finally, it is assumed that there is at least one other pathway for the detection of the dsDNA of microbes, based in part on DNA sensing in cells despite absence of the DAI pathway [25]. The known receptors for viral DNA ultimately induce interferons, cytokines, and programmed cell death pathways.

Apoptosis is the programmed death of dangerous or unnecessary cells, for example, virally infected, aging, or malignant cells. It is thus one of the most ancient forms of innate immunity. Certain cellular bcl-2 proteins mediate resistance to programmed cell death (apoptosis) [50–52], typically via interaction with proapoptotic bcl-2-related proteins [52]. Human bcl-2 also leads to increased nuclear translocation of the transcription factor, NF-*κ*B [53–55], which typically promotes cell survival [14, 56, 57]. Other cellular bcl-2 proteins promote cell death in response to harmful stimuli such as viral infection. Other effectors of programmed cell death are caspases. Cleavage of cellular caspases and/or loss of mitochondrial integrity promote cell death in the face of many stimuli including viral infection. Still other death programs include pyroptosis—the death of cells following activation of the PYRIN domains and IL-1 release. Thus, in the absence of viral innate immune evasion, apoptosis provides an antiviral mechanism for the elimination of virally infected cells.

## 3. Evolution of the Antiviral Innate Immune Response: Different Genes, Recurring Themes

Prokaryotic organisms encode primordial proteins that recognize the molecular patterns (e.g., specific sequences of DNA of bacteriophages) from pathogens (i.e., bacteriophages) and thus can be considered to possess a primitive innate immune system. Although the mechanisms of innate immunity in bacteria differ radically from those of higher organism, four principles of innate immunity are preserved in several mechanisms (Table 1). First, the clustered regularly space short palindromic repeats (CRISPERs) of bacteria and archaea encode a series of palindromic sequences that target pathogen DNA and suppress their transcription in a way similar to the antiviral action of microRNAs of *Drosophila* [58, 59]. Second, following exposure of prokaryotes to bacteriophages, the phage shock protein (Psp) signaling pathway involves an unknown sensor and signal transduction by the leucine zipper protein PspB. PsP signaling is initiated in response to loss of cell membrane integrity induced by stresses such a bacteriophage infection [60]. This is similar in principle to the enhanced cell membrane integrity mediated by interferon in the mammalian antiviral response [2, 13, 19]. A third conserved principle is intracytosolic nucleic acid recognition (analogous to mammalian RLRs or *Drosophila* DICER) that triggers an innate immune response. In bacteria, restriction endonucleases recognize and digest bacteriophage nucleic acids, while specificity of this response is maintained by bacterial methylation of its native DNA. Fourth, programmed death of bacteriophage-infected bacteria induced by the MazF protein can pre-empt spread of viral infection, as is true of proapoptotic proteins in higher organisms (reviewed in [27]). Bacteriophage mechanisms to evade the bacterial innate immune pathways include rapid mutation to generate DNA sequence diversity that evades CRISPER, acquisition of host bacterial methylases to mask restriction sites in bacteriophage DNA [26], and programmed cell death resistance [27]. A bacteriophage mechanism to evade PsP signaling has not been reported, although it is tempting to speculate that the rapid mutation observed during bacteriophage infection might avoid detection by the Psp pathway. Although the details and evolution of innate immune mechanisms in bacterial cells are highly divergent from multicellular organisms (Table 1), the principal functional attributes of innate immune recognition and viral evasion are remarkably conserved, especially in the invertebrate innate immune responses.

*Drosophila* lack an adaptive immune system; thus, they are ideal model organisms to study innate immunity since they possess a complex innate immune system (Table 1). Viral mechanisms for suppression of innate immunity in *Drosophila* have been reviewed recently and will be discussed only briefly here [29]. In contrast to mammalian cells, *Drosophila* does not encode NOD proteins. It has been suggested that *Drosophila* TLRs encoded recognize PAMPs of viruses that are tropic for *Drosophila* [61]. Unlike mammals,* Drosophila* rely heavily on RNA interference as a defense against viruses. The protein DICER2 is a helicase/endonuclease that is related to the RIG-I-like helicase of mammals [62]. DICER2 has two effector functions; the first initiates a cascade of endonucleolytic cleavage of viral RNAs that mediate gene silencing, and the second is a RIG-I-like signaling activity of DICER2 whereby DICER-2 mediates induction of the antiviral genes, such as *Vago *[62]. A distinct protein, DICER1, cleaves isolated miRNAs that subsequently suppress transcription of viral RNA just as mammalian DICER2 does [58, 59]. To evade this innate immune defense, the *Flockhouse* virus encodes the dsRNA-binding protein B2 that inhibits dsRNA recognition by DICER1 and DICER2 in *Drosophila* [30]. Furthermore, viruses inhibit the function of inhibitors of kappaB (I-*κ*B) translocation to prevent signaling initiated by *Drosophila* TLRs [61], but not the Jak/Stat antiviral defense pathway in *Drosophila*. Other viral proteins that act in signal transduction are thought to mediate the production of antiviral peptides, including the principal *Drosophila* gene induced by viral infection that is *Vago,* which encodes a 14 kilodalton cysteine-rich polypeptide [62]. *Vago* is thought to be, in principle, analogous to interferons as it is a virus-induced protein critical to control viral infection. Finally, viral innate immune evasion proteins encoded by baculovirus inhibit the function of apoptotic pathways [31, 32, 63, 64]. Although there are parallels between the principles of innate immune defense against viruses between bacteria and *Drosophila* (Table 1), the innate immune responses of *Drosophila* more closely resemble those of the mammalian antiviral innate immune response.

## 4. Viral Evasion of Host Defenses: Highlighting Critical Components of the Mammalian Innate Immune Response

### 4.1. DNA Viruses

**Figure 1:**
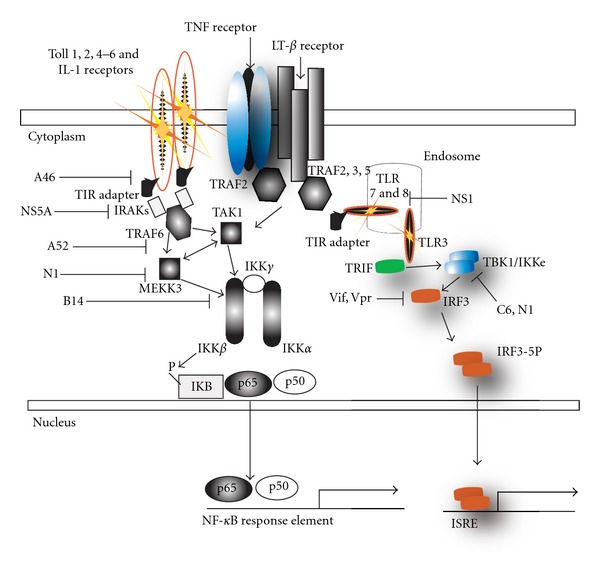
Signaling by the innate immune system that is inhibited by several viral proteins. This model depicts the salient features of TLR-induced NF-*κ*B and IRF3 induction. Several poxviral proteins N1, C6, A14 A46, and A52 inhibit the activation of NF-*κ*B and/or IRF3 signaling pathways, by interacting with and inhibiting the activity of the classical IKK complex (IKK*α*/*β*/*γ*) as well as the nonclassical IKK*ε*/TBK1 complex. HCV protein NS5A inhibits TIR signaling by MyD88, its NS3/4A digests MAVS to inhibit RLR signaling, and its core protein inhibits Jak/Stat signaling. Finally, HIV Vif and Vpr degrade IRF3.

The *Poxviridae* are large enveloped DNA viruses that replicate in the cytoplasm. Vaccinia virus (VACV) is a robust poxviral vaccine originally used to eradicate smallpox. Poxviruses encode approximately 180 genes. About 80 genes are essential for replication in tissue culture, whereas 100 encode virulence proteins, such as decoy receptors for IL-1, TNF-*α*, and interferons. These virulence proteins (Table 1, bold text, and Figure 1) interdict innate immune signaling by preventing receptor engagement at the cell surface [65]. Moreover, the pox virus proteins, **E3** and **K3**, bind dsRNA in the cytoplasm, reducing type I interferon production and, in the case of **E3**, preventing activation of the dsRNA-dependent protein kinase PKR [34].

**Figure 2:**
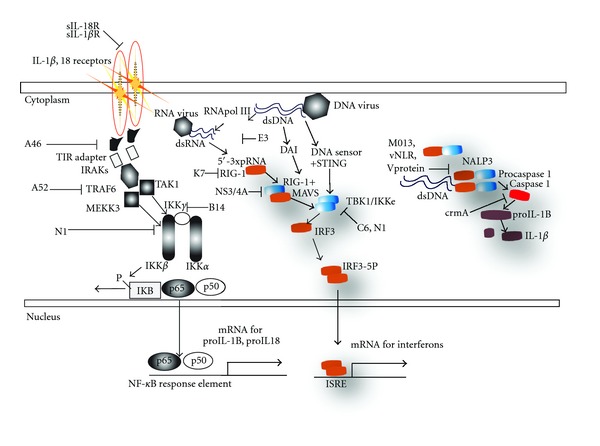
Viral proteins inhibit nucleic acid receptors of the intracytosolic innate immune response. Viruses inhibit each of the two signals that initiate the inflammasome activation process. The first signal—IL-1*β* and/or IL-18 binding and activation of the TLR/IL-1*β* receptor pathway—is inhibited by soluble IL-1*β* and IL-18 (from VACV); downstream, inhibitors of signaling to NF-*κ*B (from VACV or HCV) repeatedly target this important antiviral pathway that optimally requires NF-*κ*B translocation leading to the production of pro-IL-1*β* and pro-IL-18. Second, the inflammasome processes these pro-IL-1*β* and pro-IL-18 proteins via caspase-1 that is itself processed upon clustering mediated at the NLRP3 inflammasome upon detection of intracytosolic pathogens. This leads to IL-1*β* and IL-18 production and release that activates the IL-1*β*/IL-18 pathway in an autocrine manner, as well as the innate and adaptive immune response. Inflammasome activation is inhibited by myxoma virus M013, measles viruses V protein, and KSHV vNLR. Finally signaling to IRF3 by intracytosolic DNA or RNA is inhibited at the level of MAVS by HCV's NS3/4A and at the level of TBK1 by VACV C6 and N1 (Figure 1).

Other vaccinia virus proteins have been characterized as inhibitors of innate immune intracellular signal transduction (Figure 1). For example the VACV **N1** family of ten bcl-2 like proteins inhibits NF-*κ*B signaling [37]. Of the proteins characterized to date, N1 is the most robust VACV virulence factor, increasing replication 10,000-fold, inhibiting NF-*κ*B, IRF3 and apoptotic signaling [35, 66, 67]. **A52** inhibits NF-*κ*B and increases p38 kinase activity [68]. **A46** inhibits NF-*κ*B and IRF3 signaling [36]. And **K7** inhibits IRF3 and NF-*κ*B signaling by binding to DDX3 and preventing MAVS signaling to TBK1 [5]. It is unclear what the role of N1's antiapoptotic function is as VACV *already* encodes a vbcl-2 (F1), that, unlike N1, is critical for viral survival *in vitro*. Thus, the antiapoptotic potential of the N1 vbcl-2 reconciles the absence of cell death despite N1 inhibition of NF-*κ*B. While no direct inhibitor of the inflammasome has been detected in vaccinia virus, the poxvirus serpins **SPI-1** and **crmA **inhibit caspase 1 activity downstream of the inflammasome [69] and another poxvirus, myxoma virus encodes the **M013** PYRIN domain containing protein that inhibits signaling by the inflammasome by interrupting association of NLRP3 and ASC ([45], see Figure 2). Thus poxviruses inhibit many aspects of the two-signal inflammasome inflammatory pathway by inhibiting pro-IL-1*β* and pro-IL-18 production by the IKK pathway and cleavage by the inflammasome (Figure 2). Additionally, as outlined in Figure 1, IKK complex signaling to TNF-*α*, IFN, and other cytokines is impaired by viral innate immune evasion proteins.

The gammaherpesviruses encode proteins that highlight the role of antiapoptotic factors in innate immune evasion. Herpesviruses cause a latent, life-long infection. During latent infection herpesvirus antigens are principally present in the nucleus thereby evading recognition by the cytosolic and extranuclear membrane-associated components of the innate immune system. The apoptotic mechanisms of the innate immune system affect the elimination of herpesvirus-infected cells. Two human gammaherpesviruses, Epstein Barr Virus (EBV) and Kaposi's Sarcoma Herpesvirus (KSHV) have evolved several mechanisms that induce latent infection and thus inhibit the apoptotic innate immune response (LMP1 and LANA1). Recent studies of recombinant EBV containing deletions of the genes encoding both the antiapoptotic vbcl-2, BHRF1 [47], and a second EBV bcl-2, BALF1 [48, 70, 71], have revealed that deletion of both EBV bcl-2 homologs dramatically increases the survival of cells undergoing EBV infection [72]. Finally, the KSHV gammaherpes orf63 has been shown to encode a viral NLR (**vNLR**, Figure 2) that inhibits NLRP1, NLRP3, and NOD2 function, permitting persistent KSHV infection [45].

Viral caspase inhibitors are believed to neutralize immune responses of the host that activate the caspase pathway of apoptotic cell death (although it is logical to hypothesize that viral caspase 1 inhibitors also inhibit the inflammasome). Three different viral proteins inhibit the caspase pathway of apoptosis: (1) the serpins of the poxviruses, exemplified by crmA, a caspase inhibitor encoded by the cowpox virus genome [69], and (2) the baculovirus p35 caspase inhibitor protein [73]. Finally, the v-FLIPs, which are expressed by the gammaherpesviruses equine herpesvirus 4 and Kaposi's Sarcoma Herpesvirus (KSHV/HHV-8) [46] inhibit apoptosis by competing with caspase 8 (FLICE) for binding to the death effector domains of adaptor proteins of death receptors (reviewed by [74]). Inhibition of the activation of the caspase cascade that would otherwise follow oligomerization of death receptors prevents apoptosis induced by the cascade of proteases that eventually induce cellular self-digestion [75]. Inhibition of caspases is thus another evolutionarily conserved mechanism for viruses to avoid the apoptotic host innate immune response. In addition, vFLIPs dysregulate the function of IKK-*γ*, thereby activating the IKK complex-mediated dissociation of IkB from NF-*κ*B and subsequent NF-*κ*B signaling [76]. Activation of the IKK cascade that would otherwise promote innate immune signaling activates KSHV replication and promotes KSHV-transformed cell survival. In the case of this KSHV mechanism, signaling by the classical innate immune response pathway is perturbed by KSHV protein, allowing KSHV to escape the apoptotic host innate immune response.

### 4.2. RNA Viruses

Hepatitis C Virus (HCV) is a single-stranded, positive-sense RNA virus that encodes 10 proteins. HCV typically induces a life-long infection via successfully evading the adaptive and innate immune responses. The many HCV proteins that possess dual functions in both replication and innate immune evasion likely reflect the limited number of HCV. Surprisingly for the small size of its genome, HCV shares mechanisms of innate immune evasion with much larger DNA viruses such as the poxviruses. Poxvirus nucleoside triphosphate phosphohydrolase I (NPH-I) is absolutely essential for VACV mRNA transcription and VACV replication, yet simultaneously NPH-I inhibits the interferon response [77]. The HCV NS3/4A protease/helicase encodes a helicase that suppresses the IFN-*β* promoter independently of **NS3/4A** proteolytic destruction of innate immune signaling components such as TRIF that activate the IFN-*β* [42]. A recombinant RLR engineered to encode only a helicase domain is a dominant negative inhibitor of RLR-driven interferon (IFN)-*β* promoter activity. This dominant negative RLR lacks a signaling domain [7]. Viral helicases inherently lack signaling domains, and thus viral helicases structurally resemble a dominant negative RLR and might act as RLR antagonists. HCV innate immune evasion mechanisms also include the proteolytic destruction of MAVS by the protease component of the NS3/4A protein. The destruction of MAVS, which transduces signals from the RLRs, therefore inhibits signaling to IRF3 via TBK1, blocking the interferon response. HCV core protein expression correlates with impaired signaling of the Jak/Stat pathway to IFN-*α*/*β*, although the mechanism for this is still being defined [78]. The HCV core protein inhibits TLR signaling through its chronic stimulation of TLR2 resulting in TLR hyporesponsiveness [79], HCV core protein binds STAT1, and HCV infection leads to STAT1 degradation, which inhibits the antiviral signaling in the Jak/Stat pathway [80]. Finally HCV protein **NS5A** inhibits recruitment of IRAK to the MyD88 TIR adapter [40] and may inhibit interferon production via NS5A suppression and the phosphorylation of eIF2 by the PKR kinase [40]. Thus, HCV inhibits several types of innate immune signaling via the action of only a few proteins.

Influenza viruses are enveloped RNA viruses with a negative-sense, single-stranded segmented genome. Influenza virus is an extremely virulent respiratory pathogen, and influenza virus possesses distinct innate immune evasion mechanisms that are critical for its pathogenesis. The nonstructural protein-1 (NS1) functions to inhibit the host interferon response [81], thus inhibiting activation of IRF3 [35, 81]. Deletion **NS1** dramatically attenuates influenza viruses [35, 81]. Inhibition of RLR signaling is a critical event in the lifecycle of many viral pathogens, for example, influenza virus [82, 83]. Influenza NS1 protein inhibits RIG-I signaling to NF-*κ*B and IRF3 by inhibiting the E3 ubiquitin ligase TRIM 25 required for its function [33]. Furthermore, influenza virus NS1 binds to dsRNA that would trigger the RIG-I/MDA5 antiviral response. Finally, the influenza virus polymerase activity depends upon cellular mRNA, thereby depleting host mRNAs, which has been postulated to inhibit host antiviral gene expression [45]. Similar to herpesviruses, influenza virus undergoes replication in the nucleus minimizing detection by intracytosolic nucleic acid sensors dsRNA binding by the influenza. Thus, influenza virus appears to encode inhibitors of nucleic acid sensing, host antiviral gene expression, interferon response, dsRNA, and physical separation of signal transduction components from the innate immune sensors. These mechanisms are a recurrent theme in viral innate immune evasion.

Retroviruses are highly successful at evading innate and adaptive immune responses. The rapid evolution of HIV envelope proteins and their heavy glycosylation results in epitopes that are not conducive to an adaptive immune response. The well-studied mechanisms for retroviral immune evasion include the infection and apoptotic destruction of HIV-1- or HIV-2-infected T cells. It is interesting to note that HIV protease degrades human bcl-2 [84] and that HIV Nef, an HIV accessory' protein, induces apoptosis [85]. Thus this mechanism of HIV induction of apoptosis breaks the typical paradigm where viruses encode proteins that inhibit apoptosis (see Table 1).

HIV encodes several other “accessory” proteins that are essential for HIV infectivity and pathogenesis *in vivo*. These proteins antagonize the innate immune response in several ways. Nef mediates activation of MAPK signaling to AP-1, which is suggested to activate viral replication [86, 87]. HIV **Vif** and **Vpr **degrade IRF3, thereby inhibiting signaling to IRF3 and interferon production [88]. Thus, although inhibition of adaptive immunity by HIV is well known, innate immune evasion plays an important role in HIV pathogenesis.

## 5. Conclusion: Innate Immunoevasion—From Insight to Innovation

Many viral mechanisms have evolved to evade the immune response. Surprisingly, the general outline of innate antiviral mechanism is remarkably persistent throughout evolution, such as DNA restriction/dicing and programmed cell death; however, differences between innate immune responses of distinct organisms are often more striking and may hint at novel innate immune evasion pathways still undiscovered in mammalian virus-host interactions. Signaling to interferon resembles antiviral protein induction in *Drosophila* and in some respects, even in bacteria. The evolutionary conservation of these mechanisms suggests their study will advance understanding of viral pathogenesis and that these pathways would be worthy targets of antiviral inhibitors.

In this regard, there are several promising antiviral therapies targeting viral innate immunoevasion genes. HCV protease inhibitors have been suggested to inhibit HCV innate immunoevasion, presumably by preventing MAVS digestion [38, 89], and thereby permitting critical signaling to interferon. An *in vitro* study of the N1 vaccinia virus virulence factor and innate immune evasion protein identified chemical inhibitors of its antiapoptotic function [90]. This is surprising as N1 does not mediate cell death *in vitro*, where these inhibitors were tested [91, 92]. These findings highlight the difficulty of studying certain innate immune inhibitors *in vitro.* Nevertheless, targeting potent virulence factors of viral pathogens represent a promising and entirely new approach to antiviral drug design—beyond drugs that exclusively target viral enzymes responsible for replication. Perhaps the most promising application of these studies is in the development of highly immunogenic live vaccines that contain deletions of innate immune evasion genes outlined here. Such vaccine would have the potential to be safer and potentially more immunogenic vaccine viruses by virtue of their attenuated ability to mediate innate immune suppression.

## Figures and Tables

**Table 1a:** (a) Bacteria

Mechanism	Viral evasion strategy	Virus protein
CRISPER	Genetic variation of DNA	DNA polymerase [26]
Psp-induced signaling	Unknown (Psp genetic variation)	Unknown
Restriction/methylation	Methylation of viral target DNA	Acquired bacterial methylase [26]
Apoptosis	Lysogeny/tolerance	Phage lysis gene regulation [27]

**Table 1b:** (b) *Drosophila*

Mechanism	Evasion strategy	Viral protein
DICER1	Genetic variation of DNA	Virus encodes miRNAs [28]
*Vago*	IkB, Jak/Stat inhibitors	Viral IkB, unknown inhibitor of Jak/STAT [29]
DICER2	Binding of ds RNA	FHV-B2 protein [30]
Apoptosis	Inhibitor of apoptosis (IAP)	Baculovirus p35 [31, 32]

**Table 1c:** (c) Mammalian systems

Mechanism	Evasion strategy	Viral protein
Interferon signaling	dsRNA binding	Influenza virus NS1 [33], VACV E3 (also inhibits DNApol III DNA sensing) [34]
	Inhibition of signaling	VACV N1 family [5, 35–37], HCV NS3A/4 [38], influenza virus NS1 [33], HCV core proteins inhibit Jak/Stat signaling [39], HCV NS5A inhibits MyD88 [40], HIV Vif, and Vpr degrade IRF3 [37]
	Inhibition of IFN binding	VACV soluble IFN alpha/beta receptors [41]

Viral RNA/DNA sensing	Inhibitors of RIG-I	HCV NS3A/4 proteolytic cleavage of MAVS [38, 42]
	Inhibitors of MDA5, LGP2	Paramyxovirus V proteins [43]
	Inhibitors of DDX3	VACV K7 [5]
	Inhibitors of the AIM2/NLRP3 Inflammasome	KSHV vNLR [44], myxoma virus M013 [45]
	Inhibitors of proteins activated downstream of the AIM2/NLRP3 inflammasome	Cowpox virus crmA VACV SPI-2, sIL-1*β*R, and sIL-18R [41]
	DAI	EBV EBERmiRNAs [25]

Programmed cell death	Viral inhibitor of apoptosis (IAP)	KSHV vFLIP [46]
	Viral bcl-2s	EBV bhrf1 and balf1 [47, 48],
		KSHV orf16 [49]
	Blockade of IL-1-mediated pyroptosis	Poxvirus crmA, sIL-1*β*, sIL18 [41]

## Abbreviations

PAMP: Pathogen-associated molecular pattern
NLR: Nucleotide oligomerization domain-Like receptor
TLR: Toll-like receptor
RLR: RIG-I-like receptor
RIG-I: Retinoic acid-inducible gene-I
MDA-5: Melanoma differentiation-associated gene-5
CARD: Caspase activation and recruitment domain
MAVS: Mitochondrial antiviral signaling protein
LPS: Lipopolysaccharide
TIR: Toll/IL-1 interacting receptor
NLRP3: Nucleotide oligomerization domain-like receptor Protein 3
ASC: Apoptosis-associated speck-like protein containing caspase recruitment domain
DAI: DNA-dependent activator of interferon
STING: Stimulator of interferon genes
CRISPR: Clustered regularly sPaced short palindromic repeats
Psp: Phage shock protein
VACV: Vaccinia virus
EBV: Epstein-Barr virus
KSHV: Kaposi'ssarcoma herpesvirus
HCV: Hepatitis C virus
NPH-I: Poxvirus nucleoside triphosphate phosphohydrolase I
IFN: Interferon
NS1: Nonstructural protein 1
DDX3: DEAD/H Box 3
IKK: Inhibitor ofkappa-B kinase
TBK: TANK-binding kinase 1
IRAK: IL-1 receptor-associated kinase-1
IRF3: Interferon response factor 3
IL: Interleukin
AIM-2: Absent in melanoma-2
TRIM 25: Tripartite motif-containing protein 25.
